# The Complete Chloroplast Genome Sequences of *Salvia reflexa* and Genome Comparison: Implications for Phylogeny and Plant Invasion

**DOI:** 10.1002/ece3.74078

**Published:** 2026-08-09

**Authors:** Lina Ding, Tianhe Yang, Shuai Wang, Hanyue Zhou, Ran Xin, Qing Miao, Ping Guan, Bo Qu

**Affiliations:** ^1^ College of Bioscience and Biotechnology Shenyang Agricultural University Shenyang China

**Keywords:** chloroplast genome, hypervariable regions, invasive plant, phylogenomics, *Salvia reflexa*

## Abstract

*Salvia reflexa* is an invasive plant of global concern due to its strong invasiveness and toxicity. However, limited chloroplast genomic information has hindered a comprehensive understanding of its phylogenetic relationships and evolutionary history. To investigate the genomic features associated with invasiveness, the complete chloroplast genome of *S. reflexa* was sequenced and characterized, and a comparative analysis was conducted with other invasive and non‐invasive species of the *Salvia*. The chloroplast genome of *S. reflexa* spanned 150,629 bp and possessed a typical quadripartite structure, including 82,054 bp large single‐copy region, 17,569 bp small single‐copy region, and two inverted repeats of 25,503 bp each. A total of 133 genes were identified, consisting of 88 protein‐coding genes, 37 transfer RNA genes, and 8 ribosomal RNA genes. A total of 710 long repeats and 542 SSRs were identified across the analyzed *Salvia* chloroplast genomes. Comparative genomic analysis revealed that chloroplast genomes within *Salvia* are highly conserved, although several hypervariable regions, including *ndhC‐trnV*, *ndhF*, *rpl32‐trnL*, *rps15‐ycf1*, and *ycf1*, were identified as potential molecular markers. Selective pressure analysis indicated that most genes are under purifying selection, whereas *ycf2* shows evidence of positive selection. Furthermore, phylogenetic analysis supported that *S. reflexa* is most closely related to *S. japonica* , while clustering into different clades with the invasive plant *S. tiliifolia* , suggesting distinct evolutionary trajectories. These findings provide valuable genomic resources for species identification, phylogenetic analysis, and population genetic research in *Salvia*, contributing to a deeper understanding of its evolutionary dynamics.

## Graphical abstract

This study reports the complete chloroplast genome of *Salvia reflexa* and compares it with those of other invasive and non‐invasive *Salvia* species to explore genomic features associated with invasiveness. The chloroplast genome exhibits a typical quadripartite structure and shows high conservation within the genus, while several hypervariable regions were identified as potential molecular markers. Phylogenetic analysis indicates that *S. reflexa* is closely related to *S. japonica* , providing genomic resources for species identification and evolutionary studies of *Salvia*.

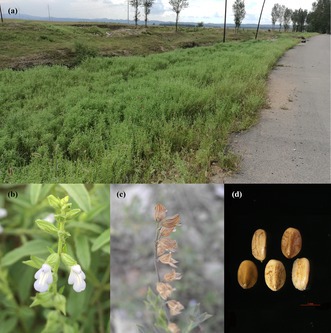

## 1 Introduction

*Salvia reflexa* is an annual herb belonging to the family Lamiaceae, native to the south‐central United States and Mexico (Williams and Hines 1939). It is characterized by high seed yield, winter dormancy, ease of germination, and strong drought tolerance. The species has spread to multiple countries, including Japan, Australia, Argentina, and China (Mito and Uesugi 2004; O'Leary and Moroni 2016; Shao et al. 2019). As an agricultural weed, it contributes to the degradation of cultivated land and soil fertility, posing a significant threat to crops such as sorghum, wheat, soybean, and sugar beet (Kegode et al. 2010; Weerakoon and Lovett 1986). Moreover, *S. reflexa* contains nitrate compounds that are toxic to livestock (Williams and Hines 1940). Current studies on this species mainly focus on toxicopathology, risk assessment, seed germination, and palynology, whereas its genetic background, invasion origin, and molecular mechanisms underlying adaptive evolution remain poorly understood (Cook et al. 2025; Gardner et al. 2021; Panter et al. 2021). To date, no studies have characterized its population genetic structure, dispersal pathways, or genes associated with environmental adaptation.

The rapid development of high‐throughput sequencing technologies has made chloroplast genome analysis a key approach to investigate invasive plants, elucidating invasion history, adaptive evolution, and phylogenetic relationships (Holt et al. 2023; Muhammad and Ki 2023; Olatinwo et al. 2025; Yang et al. 2019). Chloroplast genomes are characterized by a relatively conserved structure, moderate mutation rates, and maternal inheritance, making them reliable molecular markers for tracing invasion origins, reconstructing dispersal pathways, and identifying potentially high‐risk invasive genotypes (Corriveau and Coleman 1988; Fuentes et al. 2018; McEvoy et al. 2023). Current research in this field mainly focuses on several aspects. Whole chloroplast genome sequencing enables the characterization of genome structure, gene composition, and sequence variation, and identifies its conserved and species‐specific features in invasive plants (Kousar and Park 2023; Lubna et al. 2024). Population genetic analyses based on chloroplast DNA haplotypes and microsatellite markers can be used to infer invasion sources and dispersal routes (Maebara et al. 2020; Tao et al. 2024). Furthermore, comparative genomic analyses facilitate the identification of gene regions associated with adaptation and invasion success, such as *matK*, *ndhF*, and *clpP*, which often show relatively high divergence among closely related species and may be involved in ecological adaptation (Wang et al. 2024; Zhou et al. 2023).

The chloroplast genomes of Lamiaceae species are typically circular structure of about 150 to 200 kb in size (Yu et al. 2023). Studies on *Salvia* chloroplast genomes have largely been descriptive, and comparative analyses between invasive and native species remain limited (Gao et al. 2020; Liang et al. 2019). The chloroplast genome of *S. reflexa* , which is currently spreading rapidly in the Western Liaoning Corridor of China, has not yet been characterized. Whether this invasive species exhibits distinct chloroplast genome structures, variation patterns, or phylogenetic relationships compared with native *Salvia* species remains unclear. Such differences may be associated with its strong environmental adaptability and rapid dispersal capacity.

Therefore, this study sequenced and characterized the chloroplast genome of *S. reflexa* , along with comparative analyses of closely related species, thereby elucidating relationships among *Salvia* species. These analyses provide insights into its evolutionary relationships, genetic variation patterns, and potential adaptive mechanisms, contributing to a better understanding of its invasion biology at the genomic level.

## 2 Materials and Methods

### 2.1 Plant Materials and Chloroplast Genome Sequencing

**FIGURE 1:**
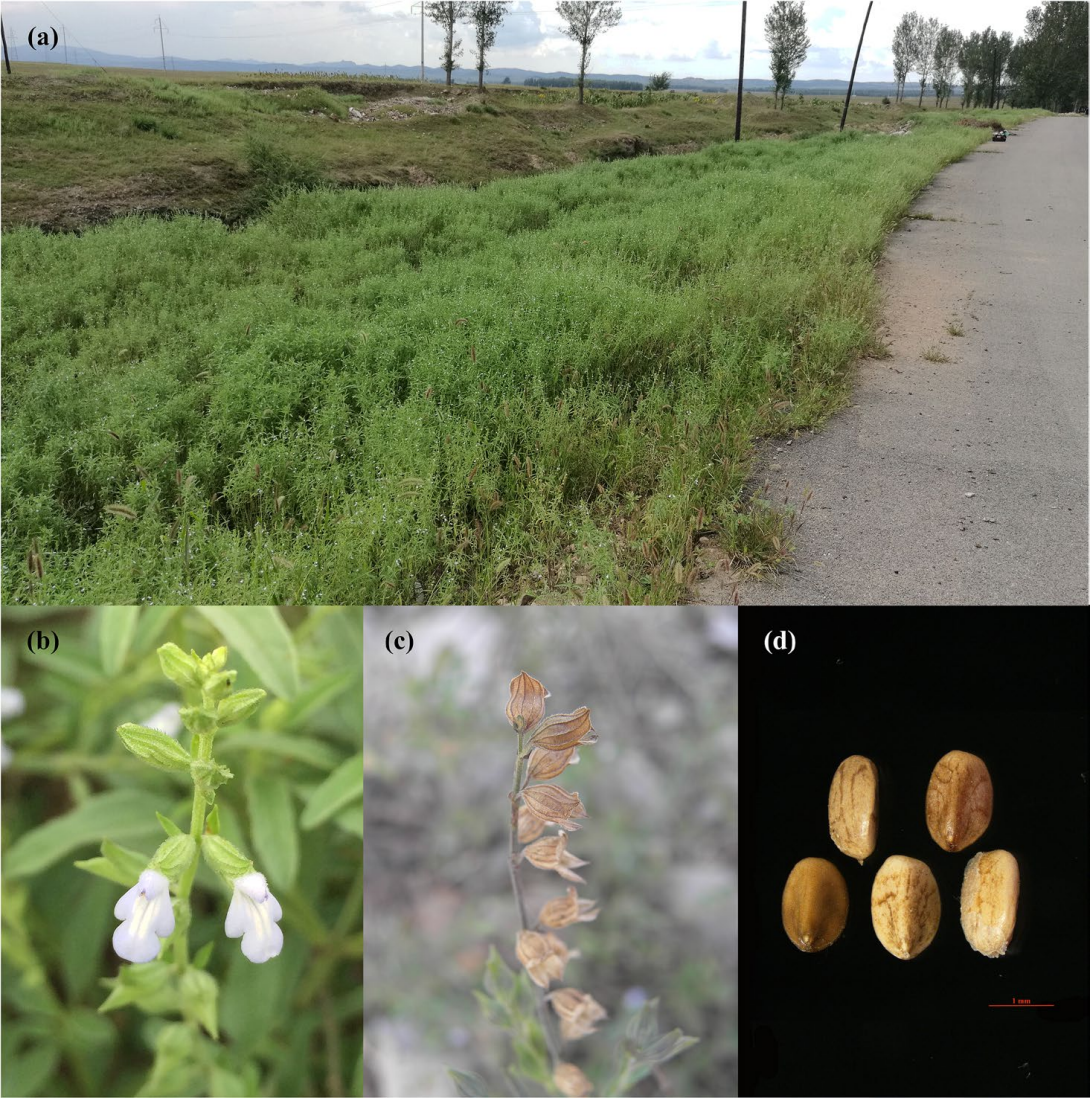
Morphological characteristics and invaded community of *Salvia reflexa* . (a) Invaded community, (b) flower, (c) persistent calyx, and (d) seed. All photographs were taken by Bo Qu and Qing Miao.

Fresh leaves of *Salvia reflexa* were collected from Lingyuan City, Chaoyang City, Liaoning Province, China (coordinates: 41.222353° N, 119.406684° E) in June 2021. The species was formally identified by Professor Bo Qu of Shenyang Agricultural University (Figure 1). The voucher specimen (Voucher code: SYAUF005919) has been deposited in the Plant Specimen Room of Shenyang Agricultural University (SYAU). Genomic DNA extraction was performed via a modified CTAB protocol (Pahlich and Gerlitz 1980). Short‐insert libraries (~400 bp) were generated following standard whole genome shotgun workflows and sequenced using paired‐end reads on an Illumina NovaSeq system at Shanghai Personal Biotechnology Co. Ltd. This process generated 3.33 GB of high‐quality clean data, with read quality monitored and verified via FastQC v.0.11.7.

### 2.2 Chloroplast Genome Assembly and Annotation

To achieve de novo assembly of the chloroplast genome, contigs were first generated from the paired‐end sequencing data and later linked into scaffolds using SPAdes v.3.11.0. Next, scaffolds relevant to the chloroplast genome were identified through reference‐guided BLAST searches against previously reported chloroplast sequences of related species; these selected scaffolds were then integrated into a consensus genome using Velvet v.1.2.07 (Zerbino and Birney 2008). The annotation of the whole chloroplast genome was performed via the online program GeSeq, with start/stop codons manually inspected and corrected (Tillich et al. 2017). Additionally, tRNAscan‐SE v.2.0.5 and GeSeq were implemented to predict tRNA genes (Lowe and Chan 2016). The final circular visualization of the genome was accomplished using OGDRAW (Lohse et al. 2007).

### 2.3 Genome Features Analysis

This study compared 15 *Salvia* species (Table 1): two invasive ( *S. reflexa* , *S. tiliifolia* ), seven native ( *S. deserta* , *S. liguliloba*, *S. przewalskii* , *S. bulleyana*, *S. miltiorrhiza* , *S. japonica* , *S. digitaloides*), and six exotic species ( *S. officinalis* , *S. hispanica* , *S. merjamie*, *S. leucantha* , *S. sclarea* , *S. lyrata* ). The total chloroplast genome length, gene count, GC content, and functional gene categories were determined using Geneious v.9.0.2 (Kearse et al. 2012). Long repeat sequences—comprising forward, reverse, palindromic, and complement types—were identified using the REPuter program (Kurtz et al. 2001) with a minimum repeat size of 30 bp and a Hamming distance of 3. To accurately quantify independent, interspersed repeats, the large‐scale global alignment between the two entire IR regions (~25 kb palindromic structure) generated by the software was excluded from the final statistical analysis. Furthermore, SSRs were identified across the 15 *Salvia* chloroplast genomes using the MISA (Beier et al. 2017). The identification thresholds were set at a minimum of 10 repeat units for mononucleotides, 5 for dinucleotides, 4 for trinucleotides, and 3 each for tetra‐, penta‐, and hexanucleotide motifs.

**TABLE 1:** Information on the 15 *Salvia* species analyzed in this study.

Species	GenBank accession no.	Native range	Status in China	Collection locality	References
*S. reflexa*	PZ179573	N. & Central USA to Mexico	E, Wi	Liaoning, China	—
*S. tiliifolia*	NC_050053	Texas to Peru	I, Ni	Yunnan, China	Wang et al. (2020)
*S. deserta*	NC_084045	Central Asia to SW. Siberia and Xinjiang	N, W/Cn	Xinjiang, China	Su, T., Hu, G.X. and Gu, L., unpublished data (2023)
*S. liguliloba*	NC_067742	China (Anhui, Zhejiang)	N, W/Cn	Zhejiang, China	Du, Yuan, et al. (2022)
*S. przewalskii*	NC_041091	Nepal to Central China	N, W/Cn	Yunnan, China	Liang et al. (2019)
*S. bulleyana*	NC_041092	China (Yunnan)	N, W/Cn	Yunnan, China	Liang et al. (2019)
*S. miltiorrhiza*	NC_020431	Central & S. China to Vietnam	N, W/Cn	Beijing, China	Qian et al. (2013)
*S. japonica*	NC_035233	S. China, Temp. E. Asia	N, W/Cn	Shaanxi, China	Yi‐Han et al. (2017)
*S. digitaloides*	NC_050895	China (Yunnan)	N, W/Cn	Yunnan, China	Zhao et al. (2020)
*S. lyrata*	NC_084048	Central & E. USA	I, Cn	Yunnan, China	Su, T., Hu, G.X. and Gu, L., unpublished data (2023)
*S. leucantha*	NC_084044	Mexico to Central America	I, Cn	Henan, China	Zhou et al. (2021)
*S. hispanica*	NC_046838	Mexico to Guatemala	I, Cn	Yunnan, China	Zhang et al. (2020)
*S. sclarea*	NC_050900	Medit. to Central Asia and W. Himalaya	I, Cn	USA	SRR8534308
*S. officinalis*	NC_038165	SW. Germany to S. Europe	I, Cn	Italy	C. Schmiderer and J. Novak, unpublished data (2018)
*S. merjamie*	NC_050897	Arabian Peninsula	A	Kenya	Zhao et al. (2020)

*Note:* Distribution status in China follows The Checklist of Cultivated Plants in China. The prefix indicates origin: N (Native), I (Introduced), E (Exotic), or A (Absent); the suffix denotes growth status: W (Wild), C (Cultivated), N (Naturalized), and W/C (both wild and cultivated). Invasiveness is indicated by appended lowercase letters: i (Invasive) or n (Non‐invasive), e.g., Ni = Naturalized and Invasive.

### 2.4 Whole Chloroplast Genome Sequence Comparisons

The chloroplast genome divergence among the 15 *Salvia* species was analyzed using the mVISTA program (Mayor et al. 2000) in Shuffle‐LAGAN mode, with the *S. japonica* genome as the reference. Genome rearrangement analyses were performed using Mauve (Darling et al. 2004) with default settings. To identify highly divergent regions within these *Salvia* chloroplast genomes, multiple sequence alignments were first conducted using MAFFT v.7.221 (Katoh and Standley 2013). Subsequently, the expansion and contraction of the IR regions were visualized using the CPJSdraw tool (Li et al. 2023) on the JSHYCloud platform (http://cloud.genepioneer.com:9929) and subsequently verified by manual inspection. Furthermore, nucleotide diversity (Pi) was calculated using DnaSP v.5.1 (Librado and Rozas 2009), with a sliding window of 600 bp and a step size of 200 bp. To validate the practical utility of the identified mutational hotspots, specific PCR primers were designed for the top three hypervariable regions (*ndhC*‐*trnV*, *rpl32*‐*trnL*, and *ycf1*) using Primer3 (Untergasser et al. 2012). The primer sequences are documented in Table S1. An initial intraspecific polymorphism test was subsequently performed using 45 *S. reflexa* individuals sampled from three geographically distinct populations in Liaoning Province, China (Figure S1). PCR amplification was carried out in a total volume of 20 μL containing 10 μL 2× Taq Master Mix, 1 μL of each primer, 1 μL of template DNA, and 7 μL of ddH_2_O. The cycling conditions were: 95°C for 5 min; followed by 30 cycles of 95°C for 30 s, 60°C for 30 s, and 72°C for 1 min; with a final extension at 72°C for 10 min. PCR products were visualized via 1% agarose gel electrophoresis and subsequently sequenced using Sanger sequencing to assess intraspecific nucleotide polymorphisms.

### 2.5 Selective Pressure Analysis

For selective pressure analysis, we constructed a *Salvia*‐focused evolutionary framework using orthologous chloroplast protein‐coding genes, with *Ocimum basilicum* serving as the reference plastome. Briefly, homologous gene sets were identified across the 15 *Salvia* chloroplast genomes, and the corresponding coding sequences were aligned using MAFFT v.7.221 (Katoh and Standley 2013). We then estimated Ka, Ks, and Ka/Ks ratios using KaKs_Calculator v.3.0 under the MLWL method (Zhang 2022). To ensure robustness, genes with NA Ka/Ks estimates, as well as those with Ka/Ks estimates equal to zero across all species, were excluded. Subsequently, genes identified with Ka/Ks > 1 were subjected to site model and branch‐site model analyses using EasyCodeML to further validate specific selective pressures (Gao et al. 2019).

### 2.6 Phylogenetic Analysis

A phylogenomic analysis involving 101 *Salvia* species was performed to ascertain the taxonomic placement of *S. reflexa* , utilizing *Prunella vulgaris* and *Callicarpa arborea* to root the trees. Phylogenies were inferred from both whole chloroplast genomes and concatenated coding sequences using Maximum Likelihood (ML) and Bayesian Inference (BI) approaches. Notably, to eliminate potential bias caused by the duplication of evolutionary signals in inverted repeats, one IR copy was excluded from each plastome sequence before alignment. The subsequent comprehensive phylogenetic analysis was entirely within the PhyloSuite platform (Zhao et al. 2025). First, a total of 80 protein‐coding genes were extracted from the chloroplast genomes using PhyloSuite's built‐in extraction tool. Using the integrated plugins within the platform, multiple sequence alignments for individual genes were executed via MAFFT under a codon‐based model, and the resulting alignments were subsequently concatenated into a single supermatrix. Thereafter, ModelFinder was implemented within PhyloSuite to identify the optimal nucleotide substitution models based on the BIC standard. Finally, ML inference was performed using the integrated IQ‐TREE plugin with 1000 bootstrap replicates, while the BI alternative was executed via the MrBayes v.3.2.2 plugin using dual parallel MCMC chains for 10,000,000 generations (sampling every 1000 generations; 25% burn‐in). Tree visualization was achieved using FigTree v.1.4, followed by esthetic enhancement via iTOL.

## 3 Results

### 3.1 General Characteristics of the Chloroplast Genomes

**FIGURE 2:**
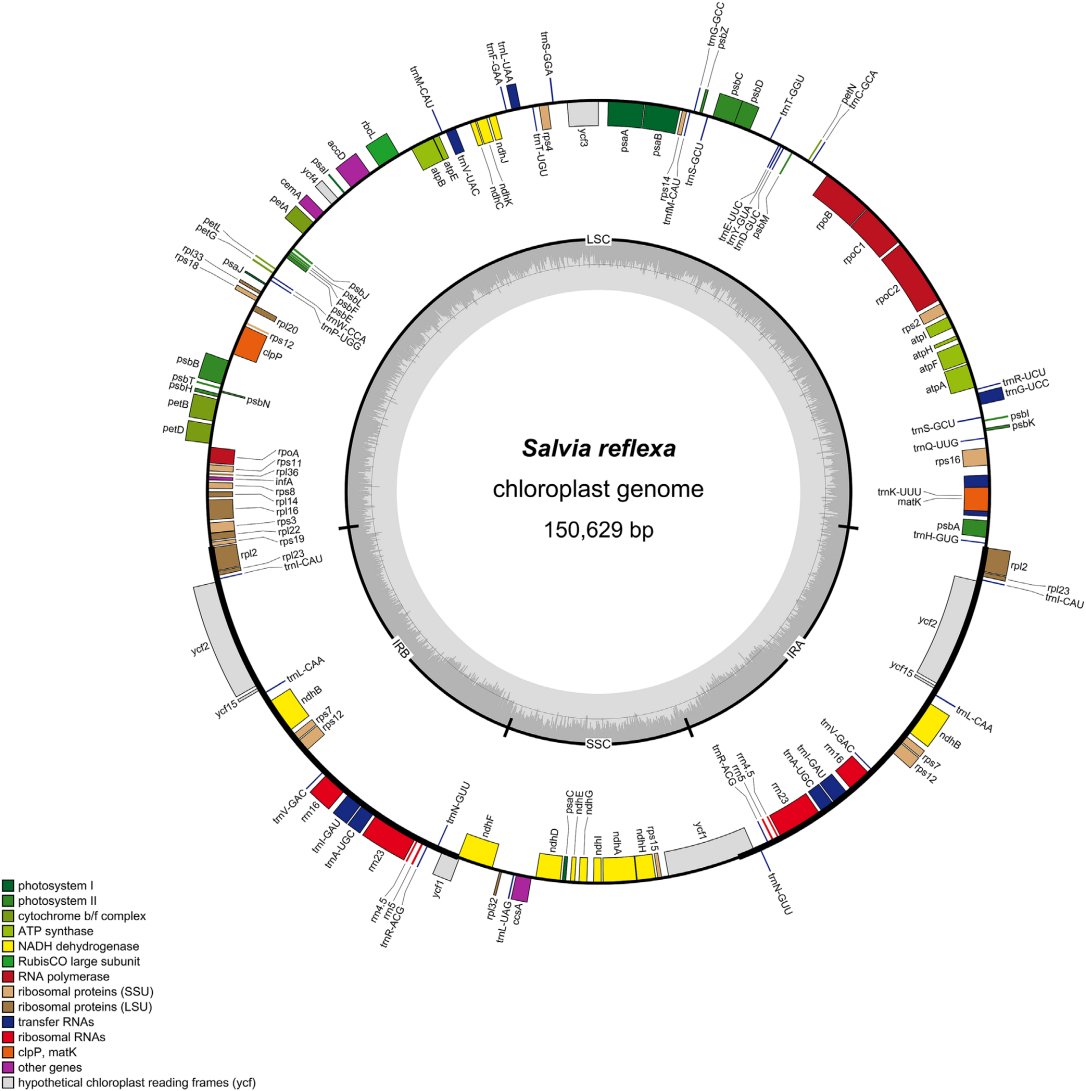
Chloroplast genome maps of *Salvia reflexa* . Genes drawn outside the outer circle are transcribed clockwise, while those insides are transcribed counter‐clockwise. Genes belonging to different functional groups are color‐coded. The dark gray in the inner circle indicates GC content of the chloroplast genomes.

The chloroplast genome map of *S. reflexa* assembled in this study is shown in (Figure 2). A characteristic quadripartite, circular architecture was observed for the chloroplast genome, which spans an aggregate length of 150,629 bp. This plastome is constituted by a large single‐copy (LSC) domain of 82,054 bp and a small single‐copy (SSC) domain of 17,569 bp, partitioned by two identical inverted repeats (IRa and IRb, 25,503 bp each). High‐throughput sequencing yielded an exceptionally high and robust coverage across the entire plastome, with an overall mean sequencing depth of 2458.69×. Specifically, the per‐region mean read depths were 2363.67× for the LSC region, 2506.85× for the SSC region, and 5158.97× and 5158.80× for the IRa and IRb regions, respectively. All genomic regions were thoroughly and sufficiently covered (well above the standard 30× threshold), with no low‐coverage gaps or ambiguities detected at the junctions between the IR and single‐copy regions (Figure S2). Comparative analysis revealed no significant differences between *S. reflexa* and the chloroplast genomes of 14 other *Salvia* species. All 15 species displayed similar GC content, with an overall GC content of approximately 38%. Within the *S. reflexa* chloroplast genome, an aggregate of 113 distinct genes was successfully characterized. This genetic complement is subdivided into 80 protein‐coding sequences, 29 transfer RNA (tRNA), and 4 ribosomal RNA (rRNA) genes (Table S2). The number and types of genes, as well as the composition of chloroplast genomes, were highly conserved across the 15 *Salvia* species (Table S3). The primary variation was observed in gene copy number, which was associated with contraction or expansion of the IR regions. For example, in *S. lyrata* , the *rpl2*, *rpl23*, and *trnI‐*CAU genes are located in the LSC region and occur as single copies, whereas in other species they are located within the IR regions and therefore present as double copies. Moreover, the genes *rps19* and *ycf1*, located at the LSC/IR and SSC/IR boundaries, respectively, were annotated as pseudogenes in certain species due to partial duplication or truncation.

### 3.2 Repeated Sequences and SSRs

**FIGURE 3:**
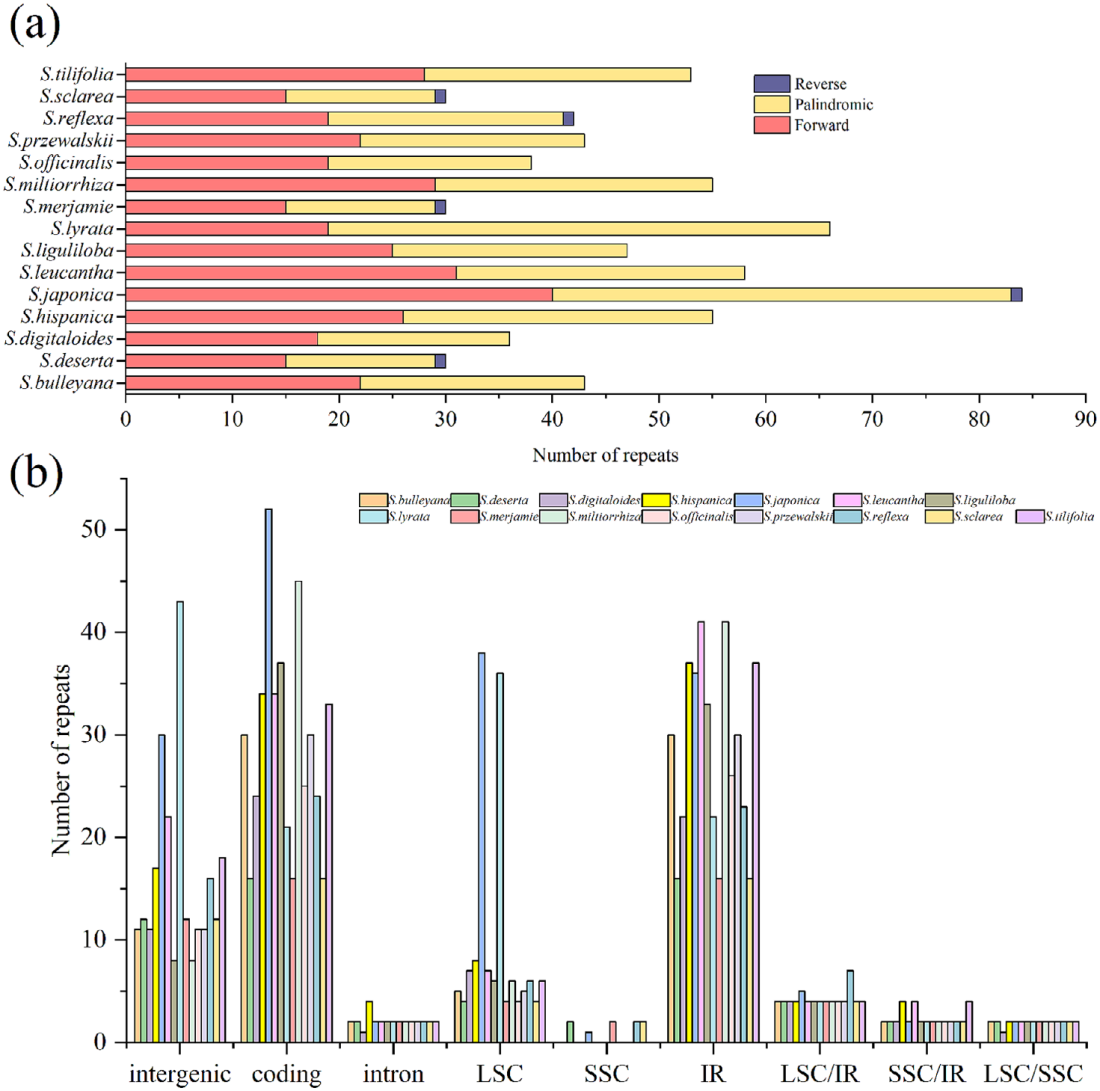
Analysis of long repeats in 15 chloroplast genomes of *Salvia*. (a) Frequency of forward, reverse, palindromic, and complement repeat. (b) Locations of the SSR in 15 chloroplast genomes.

Through REPuter analysis, an aggregate of 710 long repeats were detected within the 15 *Salvia* chloroplast genomes. The number of repeats per species ranged from 30 to 84, with the highest number observed in *S. japonica* (84), followed by *S. lyrata* (66) and *S. leucantha* (58), while *S. deserta* , *S. sclarea* , and *S. merjamie* each contained the lowest number (30). These identified repeats fall into three distinct categories: palindromic, forward, and reverse types. Palindromic repeats were the most abundant (*n* = 362; 14–47 per species), followed by forward repeats (*n* = 343) and reverse repeats (*n* = 5) (Figure 3a). Most repeat sequences ranged from 30 to 62 bp in length. However, *S. reflexa* , *S. lyrata* , and *S. japonica* contained longer repeats of 104, 94–121, and 67–410 bp, respectively (Table S4). Regarding their spatial arrangement, the LSC and SSC regions accommodate 146 and 9 repeats, respectively, while the IR domains contain 426, with an additional 129 repeats spanning across region boundaries (Figure 3b). These repeats were distributed across intergenic spacers, introns, and coding regions, with the majority located in coding sequences (437) and the fewest in introns (31) (Figure 3b).

**FIGURE 4:**
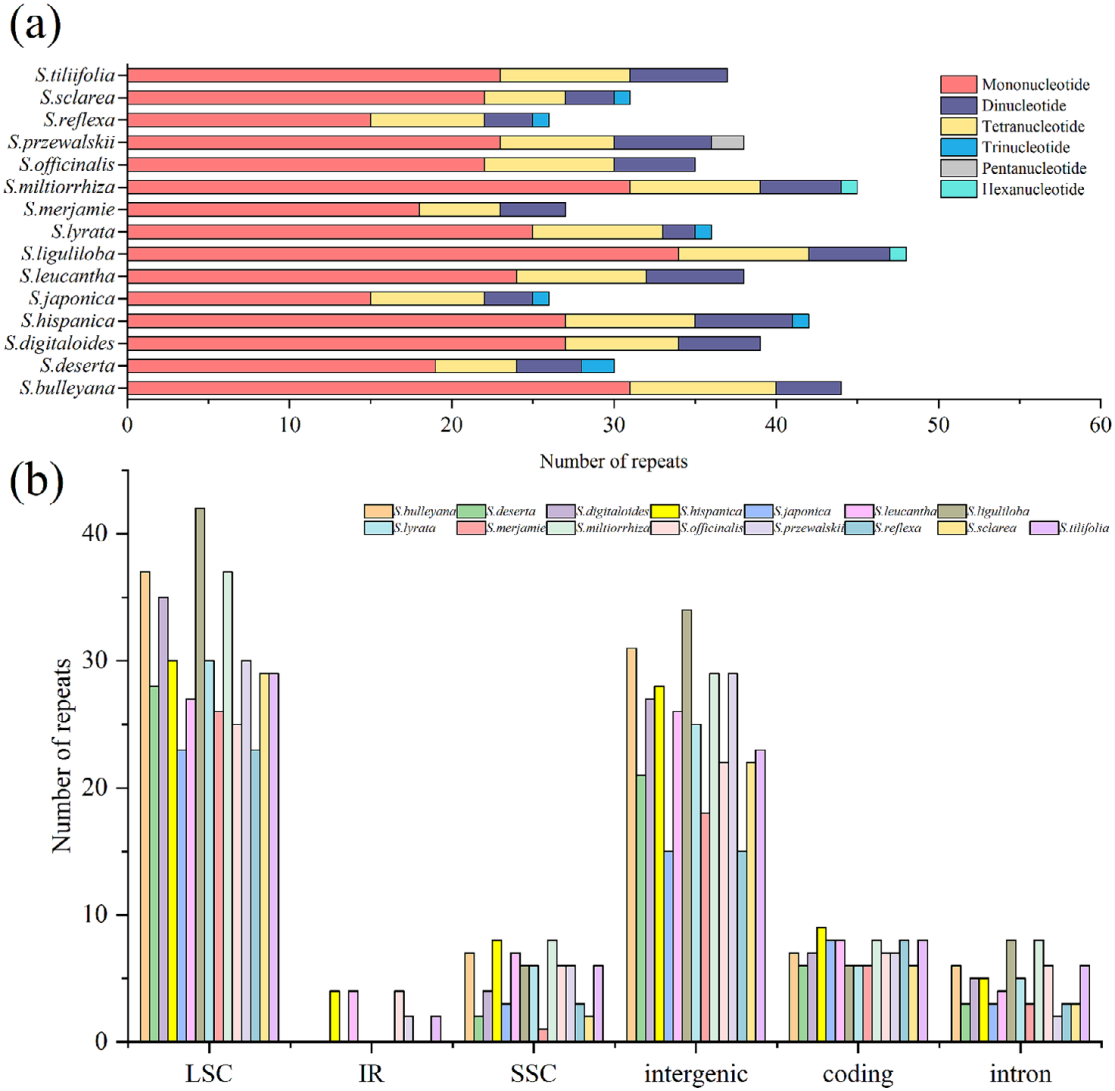
Analysis of SSRs in 15 chloroplast genomes of *Salvia*. (a) SSRs type in the chloroplast genomes of 15 species in *Salvia*. (b) Locations of SSRs.

Across the 15 *Salvia* chloroplast genomes, an aggregate of 542 SSRs were detected, with individual species frequencies spanning from a minimum of 26 (observed in *S. reflexa* and *S. japonica* ) to a maximum of 48 (in *S. liguliloba*). In terms of repeat motifs, mononucleotides dominated the repertoire (356; 65.7%), while tetranucleotides (108; 19.9%) and dinucleotides (67; 12.4%) represented the next most frequent classes. Trinucleotides (7; 1.3%), alongside pentanucleotides and hexanucleotides (each comprising 2; 0.4%), were much scarcer (Figure 4a). Of these, the majority were located in the LSC region and predominantly occurred in intergenic (Figure 4b). Most mononucleotide repeats consisted of A/T motifs (Table S5). Except for a single (C)_10_ repeat identified in *S. hispanica* , all mononucleotide SSRs were composed of A or T. Similarly, AT/TA motifs predominated among dinucleotide repeats. In terms of their spatial arrangement across the genomes, the SSRs exhibited an asymmetrical partitioning, with the LSC and SSC regions accommodating 451 and 16 markers, respectively, while the remaining 75 resided within the IR domains. These repeats were most abundant in intergenic spacer regions (365), followed by coding regions (107) and introns (70). Several species‐specific SSRs were identified, including (A)_22_ in *S. miltiorrhiza* , (ACAA)_3_ in *S. officinalis* , (AAAT)_3_ in *S. digitaloides*, (ATA)_4_ and (TTTA)_3_ in *S. lyrata* , and (A)_18_, (C)_10_, and (AAT)_4_ in *S. hispanica* . For future phylogenetic resolution and population‐level genetic assessments within *Salvia*, these polymorphic SSRs offer highly valuable molecular toolkits.

### 3.3 Expansion and Contraction of IRs

**FIGURE 5:**
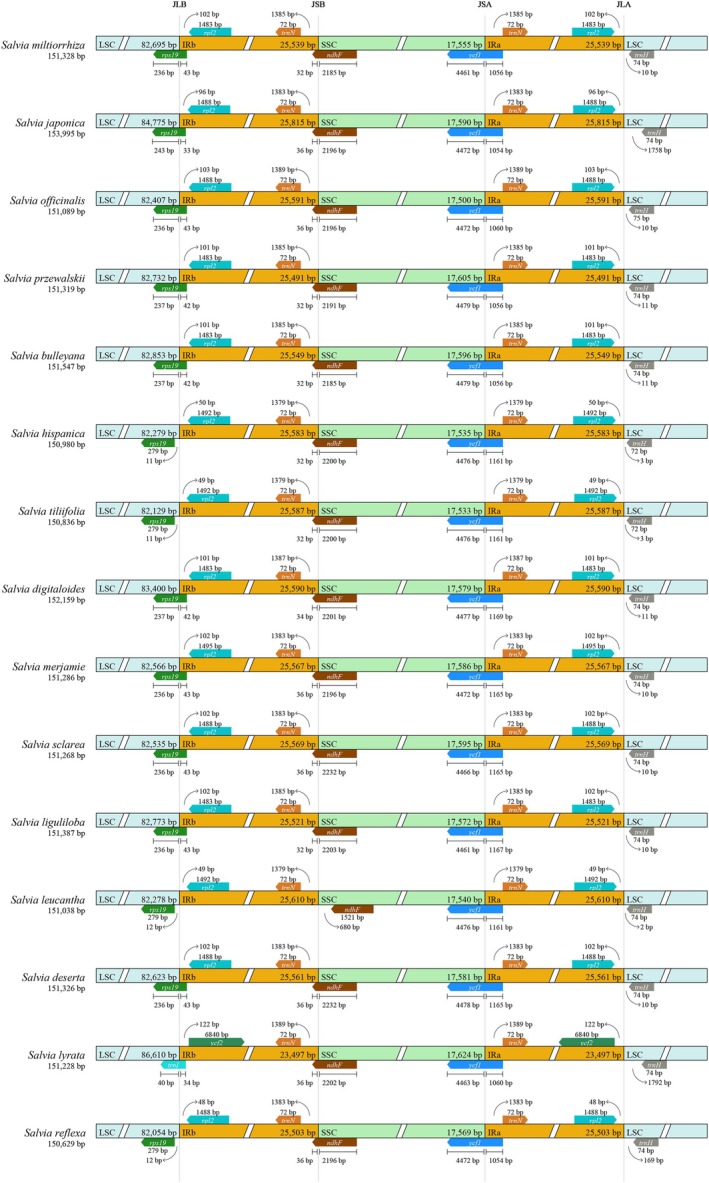
Comparison for border positions of LSC, SSC, and IR regions among the 15 *Salvia* group species. The arrow directions of the boxes with gene names indicate the direction of gene transcription. Gene lengths in the corresponding regions are displayed above or below the boxes of gene names. The number of bp represented by the arrow shows genes away from specific regions of chloroplast genome. JLB (LSC/IRb), JSB (IRb/SSC), JSA (SSC/IRa), and JLA (IRa/LSC) denote the junction sites between each corresponding two regions on the chloroplast genome.

To investigate chloroplast genomic divergence among the 15 *Salvia* species, a comparative analysis of IR boundary regions was performed. The IR regions showed minor length variation, ranging from 23,497 to 25,815 bp. Gene distribution at boundary junctions was highly conserved across all taxa (Figure 5). The *trnH* gene was consistently located in the LSC region adjacent to the LSC/IRa (JLA) boundary, while the conserved open reading frame *ycf1* spanned the SSC/IRa (JSA) junction in all species, indicating structural conservation. Variation was mainly observed at the LSC/IRb (JLB) and SSC/IRb (JSB) boundaries. The *rps19* gene, which defines the JLB boundary, crossed the junction in 10 species, including *S. miltiorrhiza* , *S. japonica* , and *S. officinalis* , *S. przewalskii* , *S. bulleyana*, *S. digitaloides*, *S. merjamie*, *S. sclarea* , *S. liguliloba*, *S. deserta* , whereas it was entirely located within the LSC region in the remaining five species ( *S. reflexa* , *S. tiliifolia* , *S. hispanica* , *S. leucantha* , and *S. lyrata* ). In *S. lyrata* , significant IR contraction led to the relocation of *rps19* into the LSC region, accompanied by the extension of the *trnI* gene across the JLB boundary. Furthermore, *rpl2* was consistently located within the IRb region in all species, while *ndhF* spanned the JSB boundary in all species except *S. leucantha* . Our evidence suggests that while the macroeconomic quadripartite conformation remains static among *Salvia* lineages, terminal fluctuations at the IR boundaries accommodate the slight variations in total nucleotide length.

### 3.4 Comparative Chloroplast Genomic Analysis

**FIGURE 6:**
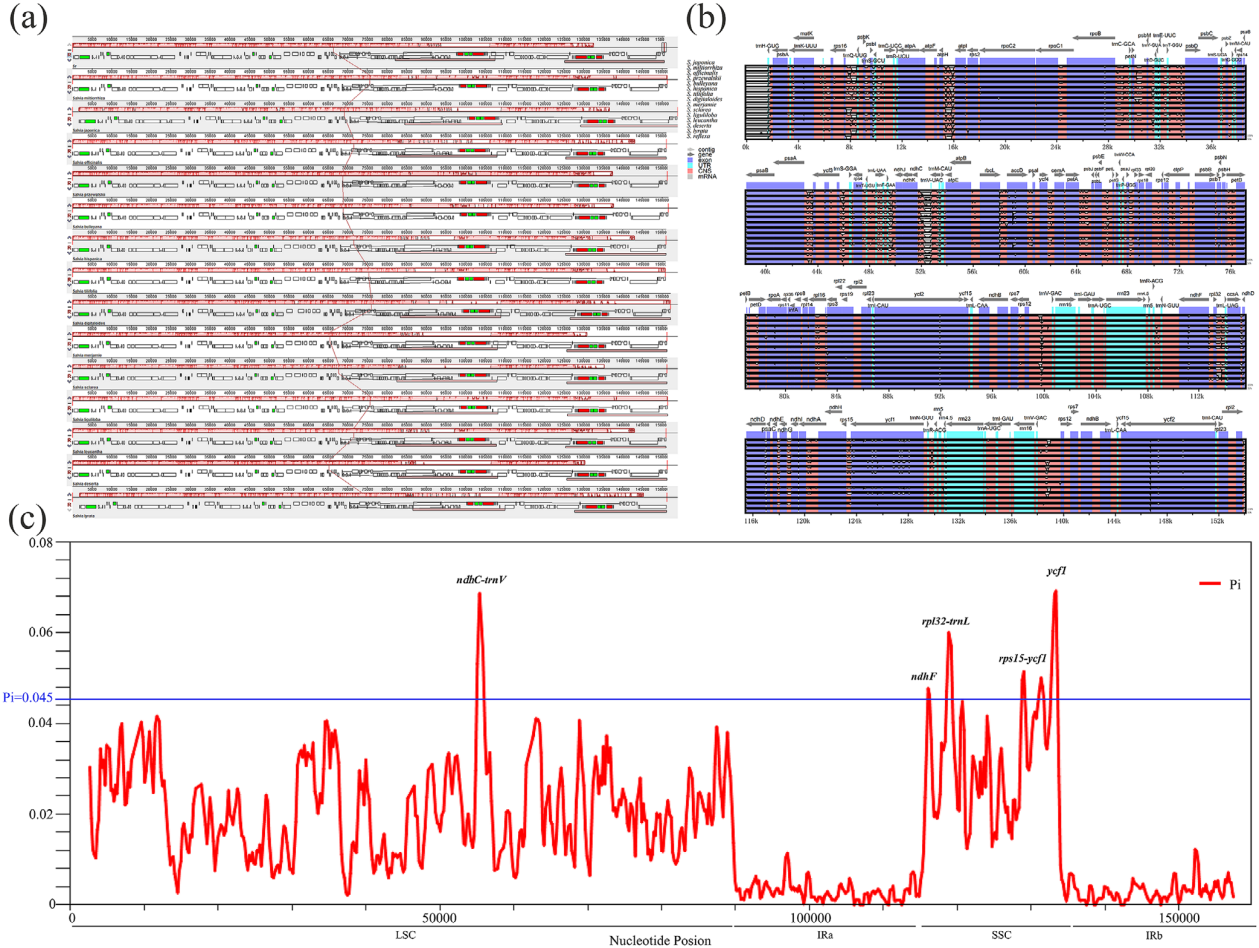
Comparative chloroplast genomic analysis. (a) Genomic rearrangement of the 15 *Salvia* chloroplast genomes. (b) Global alignment of chloroplast genomes of the 15 *Salvia* group species by mVISTA. The vertical scale indicates the percentage of identity, ranging from 50% to 100%. The horizontal axis indicated the coordinates within the chloroplast genomes. Genome regions are color coded as exton, intron untranslated regions (UTR), and conserved non‐coding sequences (CNS). (c) Comparative analysis of the nucleotide diversity values among the 15 *Salvia* group chloroplast genomes. The blue line means the Pi > 97% numerical value. The corresponding area is marked below the box.

Full‐genome alignment of the 15 *Salvia* plastomes revealed a completely synchronized gene order, establishing strict synteny and a total absence of macro‐structural rearrangements. The aligned sequences exhibited identical gene order across all taxa, confirming strict collinearity and the absence of large‐scale structural rearrangements (Figure 6a). A comparative analysis using mVISTA, with *S. japonica* as the reference, was conducted to visualize sequence identity across the 15 *Salvia* taxa (Figure 6b). The results revealed patterns of sequence conservation and divergence across the genomes. Coding regions were generally more conserved than non‐coding regions. Moreover, the LSC and SSC regions exhibited higher sequence variability than the IR regions, likely due to the stabilizing effect of gene conversion within IR sequences. Among coding genes, *accD*, *ndhF*, *ycf1*, and *ycf2* showed relatively high sequence divergence, whereas rRNA and tRNA gene regions were highly conserved.

Quantifying the genomic polymorphism surfaced in the mVISTA analysis, we estimated the nucleotide diversity (pi) across the 15 *Salvia* chloroplast lineages using DnaSP (Figure 6c), which yielded an average value of 0.016 and a maximum apex of 0.069 (Table S6). The sliding‐window trajectory confirmed that mutations were heterogeneously dispersed, with the LSC and SSC single‐copy regions displaying far greater vulnerability to sequence changes than the IR segments. Specifically, five hyper‐variable intervals—*ndhC‐trnV*, *ndhF*, *rpl32‐trnL*, *rps15‐ycf1*, and *ycf1*—emerged as undeniable mutation hotspots, each retaining a pi index greater than 0.045. To validate the polymorphism of these hotspots at the population level, we synthesized specific primers for three candidate hypervariable loci (*ndhC*‐*trnV*, *rpl32*‐*trnL*, and *ycf1*) and conducted extensive PCR and Sanger sequencing across 45 individuals from three distinct populations (Figure S3). Intriguingly, the experimental results revealed an unexpected biological phenomenon characterized by extremely low intraspecific diversity. Almost no significant sequence polymorphism was observed among the 45 individuals across the sampled populations (Figure S4), strongly indicating that *S. reflexa* in the study area has undergone a severe founder effect or a recent rapid population bottleneck—a scenario typical for invasive weeds originating from a highly limited initial gene pool. Consequently, while these regions exhibit high variability interspecifically among different *Salvia* species, their near‐fixation within the population renders them unsuitable as standard intraspecific molecular markers for *S. reflexa* .

### 3.5 Selective Pressure Analysis

**FIGURE 7:**
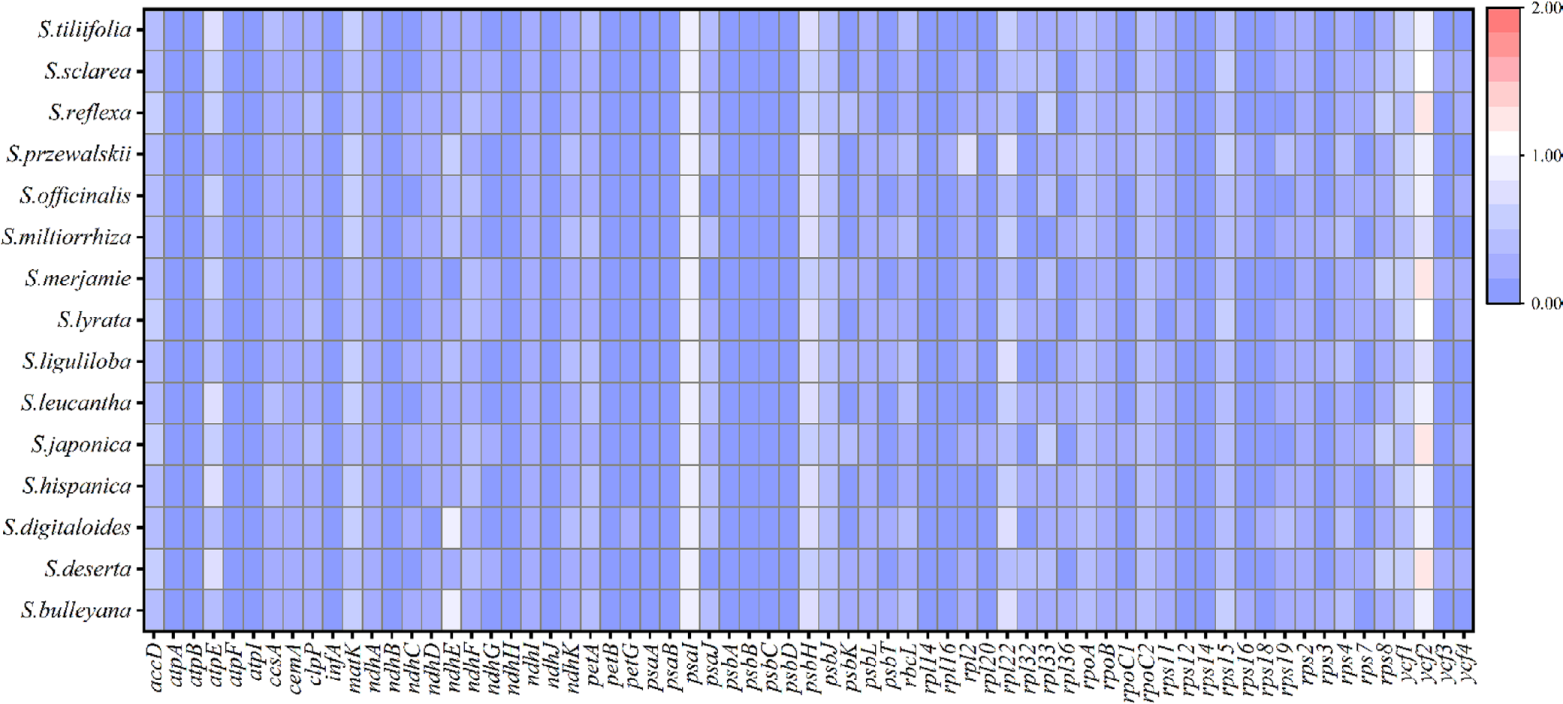
Analysis of common gene selection pressure in chloroplast genes of 15 *Salvia* species. The horizontal axis represents different species, the vertical axis represents common genes, and the color level represents the numerical value of Ka/Ks.

To investigate the adaptive evolution of chloroplast protein‐coding genes in *Salvia*, the ratios of nonsynonymous (Ka) to synonymous (Ks) substitutions (Ka/Ks) were calculated for 67 shared genes across the 15 chloroplast genomes (Figure 7).

Ka/Ks analysis revealed that 99.4% of values were < 1.0, consistent with strong purifying selection acting on most plastid genes to preserve functional integrity. By contrast, the *ycf2* gene displayed Ka/Ks ratios > 1.0 in multiple lineages, providing evidence of positive selection. This pattern was identified in six species ( *S. japonica* , *S. merjamie*, *S. sclarea* , *S. deserta* , *S. lyrata* , and *S. reflexa* ), among which *S. reflexa* and *S. japonica* exhibited the highest ratio (1.21). These results indicate that although the chloroplast genome of *Salvia* is predominantly shaped by purifying selection, certain genes, such as *ycf2*, may have undergone adaptive evolution, potentially contributing to environmental adaptation. Genes identified with Ka/Ks > 1 were subjected to site model and branch‐site model analyses using EasyCodeML to further validate specific selective pressures. The branch‐site analysis did not detect significant episodic positive selection on the pre‐defined foreground branches (*p* = 0.109 after FDR correction) (Table S7‐1). However, the site model (M8) revealed that the gene was subject to pervasive positive selection across the *Salvia* phylogeny (*p* < 0.01, LRT), successfully identifying 2 specific positively selected sites with posterior probabilities > 0.95 via the BEB approach (Table S7‐2). These results indicate that although the chloroplast genome of *Salvia* is predominantly shaped by purifying selection, certain genes, such as *ycf2*, may have undergone adaptive evolution, potentially contributing to environmental adaptation.

### 3.6 Phylogenetic Analysis

**FIGURE 8:**
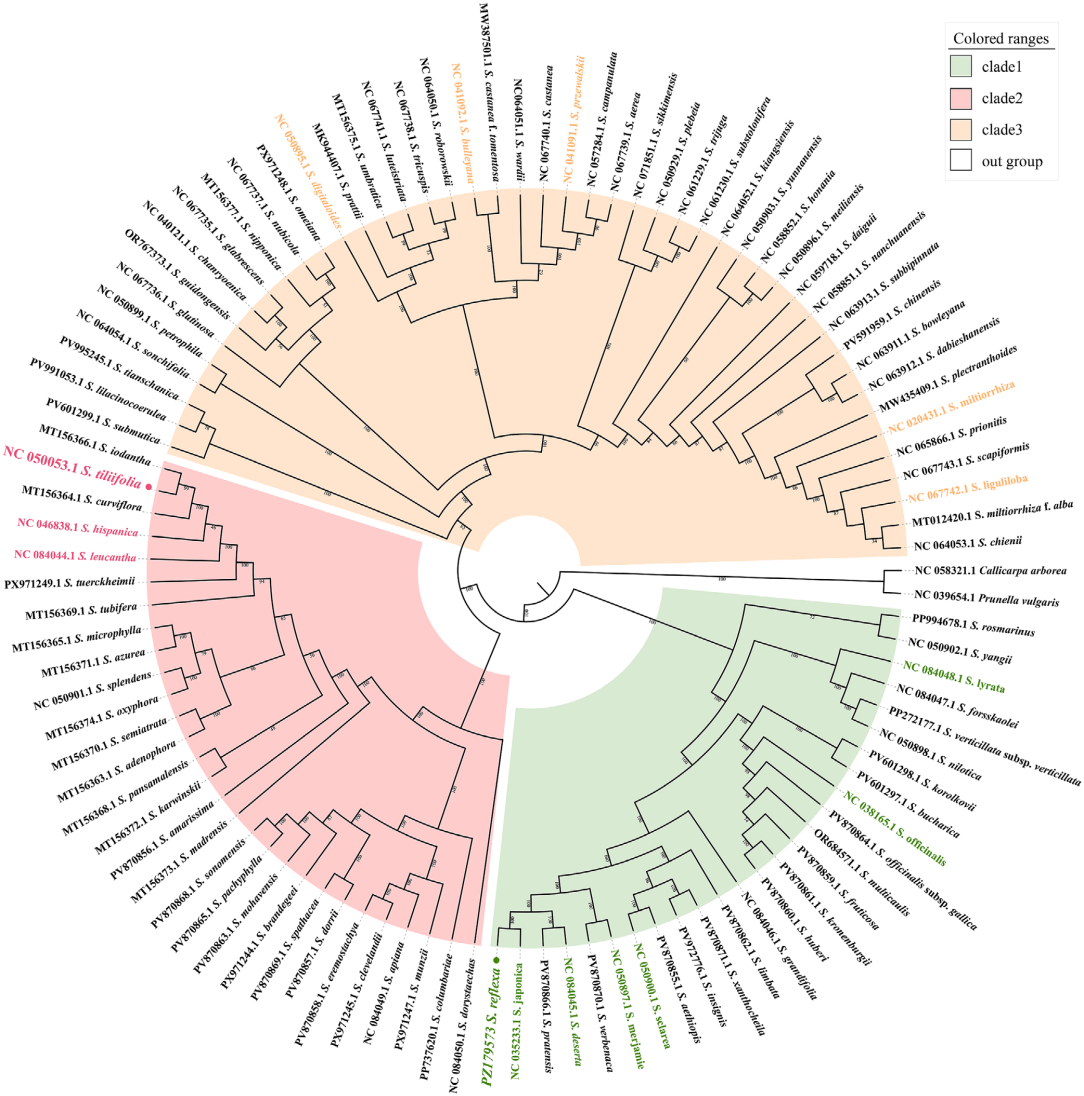
Maximum‐likelihood phylogeny of 101 *Salvia* species inferred from complete chloroplast genome sequences.

For evolutionary placement of *S. reflexa* , phylogenetic matrices were assembled from dual frameworks: 101 whole chloroplast genomes alongside 80 shared protein‐coding sequences, referenced against *Prunella vulgaris* and *Callicarpa arborea* as outgroups. Topologies derived from ML and BI frameworks demonstrated remarkable congruence between both molecular matrix types. Notably, the whole plastome sequence alignment captured enhanced cladistic resolution, yielding uniformly superior bootstrap confidence metrics and posterior probability estimations relative to the protein‐coding configuration, thereby underscoring its intensified phylogenetic signal. The phylogenomic analysis divided the 101 *Salvia* species into three well‐supported clades (Figure 8), largely consistent with traditional subgeneric classification. The two invasive species were clearly separated, with *S. reflexa* assigned to Clade 1 and *S. tiliifolia* to Clade 2, suggesting distinct evolutionary origins. Among the 15 focal species, *S. reflexa* clustered with *S. japonica* , *S. deserta* , *S. merjamie*, *S. sclarea* , *S. lyrata* , and *S. officinalis* within Clade 1. *S. tiliifolia* grouped with *S. leucantha* and *S. hispanica* in Clade 2. The remaining five native species (*S. liguliloba*, *S. przewalskii* , *S. bulleyana*, *S. miltiorrhiza* , and *S. digitaloides*) formed a well‐supported subclade within Clade 3.

## 4 Discussion

*S. reflexa* is an invasive species that has expanded rapidly in western Liaoning, China (Shao et al. 2019). This study delivers the inaugural de novo assembly of the complete *S. reflexa* chloroplast genome, offering an invaluable genomic baseline to decode the molecular mechanisms underpinning its invasive potential. By integrating this newly generated genome with published *Salvia* plastomes, particularly those of closely related species and the invasive *S. tiliifolia* , a comparative phylogenomic framework was established to identify genomic characteristics associated with invasion and to elucidate genomic signatures between invasive and non‐invasive *Salvia* lineages (Hu et al. 2013). Crucially, to properly evaluate the spatial genetic partitioning and ecological adaptation of *S. reflexa* , these compiled alignment files expedited the screening of highly polymorphic intervals and the expansion of viable genetic naming.

In general, plastome architectures inside the genus *Salvia* are characterized by pronounced structural conservation, maintaining an average length profile typically clustered between 150 and 154 kb (Du, Yang, et al. 2022). Within the sampled evolutionary lineages, the total nucleotide spectrum contracts to its minimum in *S. splendens* (150,604 bp) and reaches its maximum apex in *S. japonica* (153,995 bp). Such macro‐scale size dimorphism is predominantly modulated by length fluctuations in intergenic spacer segments, specifically clustered inside the LSC and IR intervals. Here, the newly characterized *S. reflexa* chloroplast genome encompasses 150,629 bp and shelters a standard repertoire of 133 genetic loci (88 protein‐coding, 37 tRNAs, and 8 rRNAs), aligning tightly with the genus‐wide genomic stasis documented previously (Xue et al. 2019). Comparative analysis revealed strong conservation in gene content and genomic structure among the 15 *Salvia* species. Architectural diversification was predominantly driven by polymorphism within the boundary loci *rps19* and *ycf1*, which map precisely to the LSC/IR and SSC/IR interfacial junctions, respectively. These genes are frequently annotated as pseudogenes due to partial duplication at IR boundaries, a phenomenon commonly reported in other angiosperm lineages such as *Phryma leptostachya* (Phrymaceae) and *Primulina* (Gesneriaceae) (Kim et al. 2025).

Long repeat sequences (> 30 bp) play an important role in genome evolution by promoting slipped‐strand mispairing, insertions/deletions (indels), and structural rearrangements (Xu et al. 2023). Palindromic and forward configurations constituted the primary repeat cohorts documented herein, exhibiting a spatial preference for intergenic spacers and coding exons, with a pronounced cluster inside the IR regions. Such a distribution topology mirrors the evolutionary trends captured in alternative Lamiaceae lineages, such as *Mentha aquatica* , thereby implying that repeat partitioning dynamics remain phylogenetically synchronized throughout the family (Soorni and Golchini 2025). *S. reflexa* , *S. lyrata* , and *S. japonica* exhibited relatively higher numbers of long repeats, particularly within the IR coding regions. Given their efficacy in fostering structural rearrangements, such identified sequences hold considerable promise as diagnostic genomic landmarks, providing deeper insights into population‐wide polymorphism and localized pedigree history.

SSRs are widely used as molecular markers in germplasm evaluation and phylogenetic studies due to their high polymorphism and codominant inheritance (Zhou et al. 2021; Zhu et al. 2023). In this study, the number of SSRs per genome ranged from 26 to 45, showing relatively stable abundance across species (Gao et al. 2026; Yu et al. 2023). SSRs were predominantly distributed in the LSC region, consistent with patterns reported in related genera such as *Dracocephalum* and *Isodon* (Lian et al. 2022; Yao et al. 2020). Mononucleotide repeats, particularly A/T motifs, were the most abundant, accounting for approximately 65% of total SSRs. Several species‐specific SSRs were identified, including (A)_22_ in *S. miltiorrhiza* , (ACAA)_3_ in *S. officinalis* , and (AAAT)_3_ in *S. digitaloides*, as well as unique motif combinations in *S. lyrata* and *S. hispanica* . These polymorphic SSRs constitute crucial genomic assets, offering a reliable means for untangling phylogenetic networks, monitoring population‐level genetic structures, and deciphering the evolutionary trajectories within *Salvia* lineages (Wang et al. 2022).

We further evaluated structural conservation and sequence divergence across the 15 *Salvia* chloroplast genomes. The chloroplast genome of *S. reflexa* exhibited strong syntenic conservation and collinearity with other *Salvia* plastomes, with no evidence of gene loss or large‐scale structural rearrangements (Yu et al. 2023). Structural profiling indicated that nucleotide divergence was unevenly partitioned across the plastomes, with the single‐copy domains (LSC and SSC) exhibiting accelerated evolutionary erosion than the stable IR zones. Concurrently, genomic polymorphism was heavily concentrated within intergenic regions rather than conserved exons (Gao et al. 2026; Qin et al. 2024). Although IR regions are generally considered stable, their expansion and contraction remain important drivers of genome size variation (Chen et al. 2023; Liu et al. 2021). While most species retained conserved boundary structures, a significant IR contraction was observed in *S. lyrata* , resulting in the relocation of *rps19* into the LSC region and extension of *trnI* across the JLB boundary. These boundary shifts provide useful genomic features for phylogenetic and taxonomic analyses within *Salvia* and related Lamiaceae species.

Sliding window analysis identified *ndhC‐trnV*, *ndhF*, *rpl32‐trnL*, *rps15‐ycf1*, and *ycf1* as highly variable regions within the genus. Several commonly used barcoding loci, such as *ycf1* and *ndhF*, exhibited relatively high nucleotide diversity (Pi > 0.045), indicating their potential utility for resolving phylogenetic relationships (Amar 2020; Li et al. 2021). In particular, *ndhC‐trnV* and *ycf1* were confirmed as divergence hotspots, consistent with findings in other angiosperms (Dong et al. 2012, 2015). While these loci hold substantial promise as polymorphic markers for *Salvia* phylogenetics and species identification, scaling the analysis to the population level revealed negligible sequence polymorphism among the 45 individuals across three populations. This drastic reduction in genetic diversity strongly implies a severe founder effect or a recent population bottleneck, a genomic signature typical of invasive weeds originating from a restricted gene pool (Cao et al. 2026; Guo et al. 2018; Liu et al. 2024), thereby limiting their utility for intraspecific population genetics in *S. reflexa* . Crucially, although the Sanger sequencing reads of these regions exhibited high consistency among the analyzed individuals, they collectively presented multiple InDels and mutations when aligned against our assembled NGS reference plastome. Given the successful anchoring of primers in conserved exons, we hypothesize that this systematic discrepancy might be associated with several potential biological or technical factors rather than reflecting true intraspecific variation. First, standard PCR could potentially co‐amplify a common, highly conserved lineage of nuclear plastid DNA (NUPTs) across individuals, which accumulated mutations under relaxed selection after transferring to the nucleus (Huang et al. 2017; Rousseau‐Gueutin et al. 2011). Second, as these loci (particularly *ycf1* and r*pl32‐trnL*) reside within or adjacent to the SSC region, structural inversions (isomerism) or regional assembly complexities in the reference plastome may introduce confounding alignments (Palmer 1983; Walker et al. 2015). Additionally, high‐AT homopolymer runs prevalent in these spacers might have induced systematic Taq polymerase slippage during Sanger sequencing (Kelchner 2000), creating artificial InDel shifts contrasting with our high‐depth NGS assembly. Ultimately, the precise underlying mechanisms for these discrepancies remain open to further experimental verification.

For a comprehensive screening of selective profiles acting on the chloroplast protein‐coding sequences, Ka/Ks ratios were calculated for 67 orthologous genes across the 15 genomes. Most genes (99.4%) exhibited Ka/Ks values < 1.0, indicating strong purifying selection and functional conservation of the plastid genome (Nei and Kumar 2000). Contrasting with this pervasive evolutionary stasis, the *ycf2* gene emerged as a prominent evolutionary anomaly, exhibiting distinct signatures of positive selection (Ka/Ks > 1.0) in six species, and peaking at 1.21 in both *S. reflexa* and *S. japonica* . Given that *ycf2* encodes an essential subunit of the 2‐MDa chloroplast inner membrane translocon (TIC) complex mediating polypeptide import (Xing et al. 2025), its adaptive signatures were further interrogated via codon substitution models. Interestingly, while the branch‐site analysis failed to detect significant episodic positive selection on pre‐defined foreground branches (*p* = 0.109 after FDR correction), the site model (M8) revealed pervasive positive selection among the 15 sampled *Salvia* taxa (*p* < 0.01, LRT), successfully identifying 2 specific adaptive sites with high posterior probabilities (> 0.95) via the BEB approach. This divergence between model outcomes strongly implies that the adaptive evolution of *ycf2* within the studied cohort is driven by widespread, site‐specific functional fine‐tuning rather than lineage‐specific evolutionary bursts. Consequently, these identified sites likely represent crucial structural nodes undergoing conformational modifications to optimize protein import kinetics or enhance membrane proteostasis under selective pressures (Yu et al. 2023; Zhao et al. 2021; Zhu et al. 2023), although whether this adaptive pattern remains universal across the entire *Salvia* genus warrants further investigation with broader taxonomic sampling. This localized evolutionary dynamism within a highly conserved genomic blueprint provides a vital genetic prerequisite for ecological resilience, potentially facilitating the colonization of *S. reflexa* across heterogeneous environmental niches and fueling its invasive potential.

Chloroplast phylogenomics provides an effective framework for elucidating both the evolutionary history of *Salvia* and the biogeographic processes underlying its diversification and invasion (Gao et al. 2026; Muhammad and Ki 2023; Yang et al. 2019; Yu et al. 2023). In this study, phylogenetic trees constructed using 80 protein‐coding genes and complete chloroplast genomes showed largely consistent topologies. However, the complete plastome dataset provided higher statistical support for most nodes, likely due to the inclusion of additional phylogenetic signals from non‐coding regions, which showed higher sequence variability in the sliding‐window analysis. These results highlight the advantage of whole plastome data for resolving complex evolutionary relationships in rapidly diversifying plant groups such as *Salvia*. Integrating phylogenetic relationships with invasive status and geographic origin suggests distinct eco‐evolutionary patterns among *Salvia* species. The invasive species *S. reflexa* , native to semi‐arid regions of North America, likely evolved under environmental pressures such as limited precipitation, poor soil conditions, and temperature fluctuations (Shao et al. 2019). These conditions may have contributed to the development of high environmental tolerance and ecological plasticity, facilitating its successful establishment in new habitats. Whereas, *S. tiliifolia* , native to seasonally shaded areas from Texas to Peru, exhibits broad climatic adaptability that may support its spread across ecologically analogous habitats (Nyssen et al. 2025). Many native species, including *S. deserta* , *S. liguliloba*, *S. przewalskii* , and *S. miltiorrhiza* , are largely restricted to China and Central Asia. These species appear to have evolved under relatively stable environmental conditions, resulting in strong local adaptation but limited dispersal potential. Cultivated species such as *S. officinalis* , *S. hispanica* , and *S. leucantha* show a different pattern. Although native to Europe or Central America, they have been widely introduced for medicinal, edible, or ornamental purposes, leading to significant range expansion driven by human activity. Finally, some non‐invasive species with relatively broad natural distributions, such as *S. sclarea* , *S. merjamie*, and *S. lyrata* , do not exhibit invasive behavior. This may be due to factors such as ecological specialization, reproductive constraints, or limited competitive ability.

The plastome‐based phylogenetic framework established in this study improves the resolution of evolutionary relationships within *Salvia* and suggests potential links between evolutionary history, ecological adaptation, geographic origin, and invasion potential. These findings provide a molecular basis for understanding diversification patterns within the genus and offer insights for assessing ecological risks associated with invasive species. However, the phylogenomic analysis was based solely on chloroplast genome data, which reflect maternal inheritance and may not fully capture the evolutionary history shaped by hybridization, introgression, or incomplete lineage sorting. Integrating nuclear and mitochondrial genomic data would help resolve potential phylogenetic inconsistencies and provide a more robust evolutionary framework.

## 5 Conclusion

To conclude, the evolutionary drivers and genomic divergence shaping this taxonomically challenging group are effectively elucidated here through the preliminary sequencing of the *S. reflexa* chloroplast blueprint and a multi‐species comparative audit encompassing 15 *Salvia* representatives. While maintaining a stable quadripartite structural configuration and high genomic synteny, the major variations in overall sequence length across these chloroplast genomes were mathematically driven by the micro‐evolutionary expansion and recession of the IR borders. Bioinformatics mining yielded a genomic repository encompassing 710 long‐fragment repeats and 542 individual SSRs, offering reliable data assets to support down‐stream population‐level screening and diagnostic DNA barcoding assays. Several highly variable regions, including *ndhC‐trnV*, *ndhF*, *rpl32‐trnL*, *rps15‐ycf1*, and *ycf1*, were identified as mutation hotspots in inter‐specific comparisons of chloroplast genomes, and the *ycf2* gene showed evidence of positive selection, suggesting localized adaptive evolution within the plastome. Phylogenomic analysis based on complete chloroplast genomes produced a well‐supported evolutionary framework that elucidates relationships among *Salvia* species. To advance downstream initiatives in phylogenetic placement and contemporary invasion ecology, the newly decoded *Salvia* datasets serve as an indispensable informational treasury, providing crucial molecular reference thresholds to trace the evolutionary trajectories and adaptive mechanisms underlying successful biological invasions.

## Author Contributions

**Lina Ding:** data curation (equal), formal analysis (equal), software (equal), visualization (equal), writing – original draft (equal). **Tianhe Yang:** data curation (equal), software (equal), writing – original draft (equal). **Shuai Wang:** data curation (equal), investigation (equal), visualization (equal). **Hanyue Zhou:** data curation (equal), investigation (equal). **Ran Xin:** data curation (equal), investigation (equal). **Qing Miao:** data curation (equal), investigation (equal), resources (equal). **Ping Guan:** conceptualization (equal), methodology (equal), project administration (equal), writing – review and editing (equal). **Bo Qu:** conceptualization (equal), formal analysis (equal), investigation (equal), methodology (equal), project administration (equal), supervision (equal), writing – review and editing (equal).

## Conflicts of Interest

The authors declare no conflicts of interest.

## Supporting information


**Figure S1:** Geographic locations and field photographs of the study sites. (a) Map of the sampling sites; (b) Field photograph of the Jianping sampling site; (c, d) Sampling map and recent plant community characteristics of the Lingyuan site; (e) Field photograph of the Fumeng sampling site.
**Figure S2:** Read mapping and coverage depth analysis of the *Salvia reflexa* chloroplast genome.
**Figure S3:** PCR gel electrophoresis validation of three chloroplast highly variable regions across different populations. (a) Amplification of the *ndhC*_*trnV* region; (b) Amplification of the *rpl32*_*trnL* region; (c) Amplification of the *ycf1* region.
**Figure S4:** Multiple sequence alignments of three highly variable regions (*ndhC*_*trnV*, *rpl32*_*trnL*, and *ycf1*) among 45 *Salvia reflexa* individuals from three populations. (a, c, e) Alignments of the 45 PCR sequencing results against the corresponding reference segments from the de novo assembled chloroplast genome for *ndhC_trnV* (a), *rpl32_trnL* (c), and *ycf1* (e). (b, d, f) Multiple sequence alignments among the 45 PCR sequencing segments themselves for *ndhC_trnV* (b), *rpl32_trnL* (d), and *ycf1* (f).

**Table S1:** Primer sequences of the three hypervariable regions in *Salvia reflexa* .

**Table S2:** General characteristics of the chloroplast genomes of the 15 *Salvia* species.

**Table S3:** Distribution information of genes for 15 species in *Salvia* species.

**Table S4:** Complex repeats (> 30 bp) identified within the chloroplast genome of 15 species in *Salvia* species.

**Table S5:** Total number of SSRs identified within the chloroplast genome of 15 species in *Salvia* species.

**Table S6:** The nucleotide variability (*π*) value of 15 *Salvia* species.

**Table S7:** Positive selection analysis of the chloroplast genome based on branch‐site model and site model.

## Data Availability

The complete chloroplast genomes of *Salvia reflexa* were submitted to GenBank under accession numbers PZ179573. The raw data were submitted to the Sequence Read Archive of NCBI with a BioProject accession of PRJNA1479208, BioSample accession of SAMN60945440, and SRA accession of SRR39197169.
