# Supplementary material for: The Complete Chloroplast Genome Sequences of Salvia reflexa and Genome Comparison: Implications for Phylogeny and Plant Invasion
Source: Ecol Evol. 2026 Aug 9;16(8):e74078. doi: 10.1002/ece3.74078 (PMC13454378; doi:10.1002/ece3.74078)
Supplement: Supplementary file 1 [file ECE3-16-e74078-s007.docx]

**The complete chloroplast genome sequences of *Salvia reflexa* and genome comparison: Implications for phylogeny and plant invasion**

Lina Ding^1 a^, Tianhe Yang^1 a^, Shuai Wang^1^, Hanyue Zhou^1^, Ran Xin^1^, Qing Miao^1^, Ping Guan^1*^, Bo Qu^1*^

^1^ College of Bioscience and Biotechnology, Shenyang Agricultural University, Shenyang 110161, China

Author information

* Corresponding author:

Bo Qu

Address: 120 Dongling Road, Shenhe District, Shenyang, Liaoning, 110161

Tel: +86 15004099009

E-mial: syau_qb@163.com

Ping Guan

Address: 120 Dongling Road, Shenhe District, Shenyang, Liaoning, 110161

Tel: +86 18742407720

E-mial: 18742407720@163.com

^a^ Lina Ding and Tianhe Yang contributed equally to this work

**Supporting Information**

Figure S1. Geographic locations and field photographs of the study sites. (a) Map of the sampling sites; (b) Field photograph of the Jianping sampling site; (c, d) Sampling map and recent plant community characteristics of the Lingyuan site; (e) Field photograph of the Fumeng sampling site.

Figure S2. Read mapping and coverage depth analysis of the *Salvia reflexa* chloroplast genome.

Figure S3. PCR gel electrophoresis validation of three chloroplast highly variable regions across different populations. (a) Amplification of the *ndhC*_*trnV* region; (b) Amplification of the *rpl32*_*trnL* region; (c) Amplification of the *ycf1* region.

Figure S4. Multiple sequence alignments of three highly variable regions (*ndhC*_*trnV*, *rpl32*_*trnL*, and *ycf1*) among 45 S*alvia reflexa* individuals from three populations. (a, c, e) Alignments of the 45 PCR sequencing results against the corresponding reference segments from the de novo assembled chloroplast genome for *ndhC_trnV* (a), *rpl32_trnL* (c), and *ycf1* (e). (b, d, f) Multiple sequence alignments among the 45 PCR sequencing segments themselves for *ndhC_trnV* (b), *rpl32_trnL* (d), and *ycf1* (f).

Figure S1. Geographic locations and field photographs of the study sites. (a) Map of the sampling sites; (b) Field photograph of the Jianping sampling site; (c, d) Sampling map and recent plant community characteristics of the Lingyuan site; (e) Field photograph of the Fumeng sampling site. Abbreviations: JP, Jianping County, Chaoyang city, Liaoning, China; LY, Lingyuan city, Chaoyang, city Liaoning, China; FM, Fuxin Mongolian Autonomous County, Fuxin, Liaoning, China.

Figure S2. Read mapping and coverage depth analysis of the *Salvia reflexa* chloroplast genome.

Figure S3. PCR gel electrophoresis validation of three chloroplast highly variable regions across different populations. (a) Amplification of the *ndhC*_*trnV* region; (b) Amplification of the *rpl32*_*trnL* region; (c) Amplification of the *ycf1* region. For each gel plot, 49 lanes are loaded from left to right as follows: Lanes 1, 26, 27, and 49 represent the 2,000 bp DNA ladder (Marker); Lanes 2-16 contain 15 individual samples from the LY; Lanes 17-25 and 28-33 contain 15 individual samples from the JP; Lanes 34-48 contain 15 individual samples from the FM.

Figure S4. Multiple sequence alignments of three highly variable regions (ndhC_trnV, rpl32_trnL, and ycf1) among 45 *Salvia reflexa* individuals from three populations. (a, c, e) Alignments of the 45 PCR sequencing results against the corresponding reference segments from the de novo assembled chloroplast genome for *ndhC*_*trnV* (a), *rpl32*_*trnL* (c), and *ycf1* (e). (b, d, f) Multiple sequence alignments among the 45 PCR sequencing segments themselves for *ndhC*_*trnV* (b), *rpl32*_*trnL* (d), and *ycf1* (f). For each alignment plot, sequences 1-15 are derived from the LY, sequences 16-30 from the JP, and sequences 31-45 from the FM. black bars indicate nucleotide variations, while horizontal lines represent gaps.
